# Inpatient Rehabilitation is Effective for Severe Daily Activity Deficits Related to Chronic Low Back Pain

**DOI:** 10.5041/RMMJ.10530

**Published:** 2024-10-28

**Authors:** Elena Aidinoff, Sharona Yosef Ayalon, Dianne Michaeli, Ilana Gelernter, Amiram Catz, Vadim Bluvshtein

**Affiliations:** 1 Department of Spinal Rehabilitation, Loewenstein Rehabilitation Medical Center, Ra’anana, Israel; 2 Department of Rehabilitation and the Statistical Laboratory, Tel Aviv University, Tel Aviv, Israel

**Keywords:** Activities of daily living, back pain, disability, outcome assessment, rehabilitation

## Abstract

**Background and Objective:**

Chronic low back pain disability (CLBPD) is a syndrome that includes pain, disability, emotional impairments, and social problems. The study was conducted to examine the effect of an inpatient rehabilitation program on the performance of everyday life tasks (daily activities), and report on pain in CLBPD patients with primary activities of daily living (ADL) deficits.

**Methods:**

In a retrospective cohort study, demographic and clinical data were retrieved from records of inpatients admitted consecutively to the program. Scores of the Spinal Pain Independence Measure (SPIM) and of changes in reported pain levels were used to assess improvement in the performance of daily activities and pain reduction. *T*-tests were used to assess the significance of score changes. Spearman’s correlations and analysis of variance were used to assess relationships of SPIM gain and affecting factors.

**Results:**

Ninety-nine patients were included. Daily task performance improved in 71 patients (71.7%). The SPIM score increased from 48.7 (SD 16.3) at admission to the rehabilitation program to 57.8 (SD 12.5) at discharge (*P*<0.001). The SPIM score at admission negatively affected SPIM gain (*P*<0.001). The SPIM gain was significant for admission SPIM scores of 50 or lower (*P*<0.05), but differences in SPIM scores were not as noticeable for patients with admission SPIM scores above 50. Relief in pain was reported in 59 patients (59.6%) and was not associated with function.

**Conclusions:**

The multidisciplinary rehabilitation program, which improved function and provided limited pain relief in inpatients with CLBPD primary ADL deficits, can be effective for the most severe CLBPD cases.

## INTRODUCTION

Low back pain (LBP) is a severe and relatively frequent medical, economic, and social problem.^1^,^2^ In the majority of LBP patients, the source of pain is not detected, and the condition is addressed as non-specific LBP.^3^,^4^ Chronic LBP is when LBP lasts longer than three months. When LBP interferes with activities of daily living (ADL) or employment, it is referred to as chronic low back pain disability (CLBPD). Persistent pain, depression, anxiety, and domestic and social problems are among the components of CLBPD, as are difficulties in performing daily activities and in participating in social activities and in the labor force.^1^,^5^–^8^

The cause of CLBPD is probably related to a combination of physiological changes as well as social and psychological issues, such as distress, exaggerated fear of pain, and avoidance of activity. The consequences of pain, such as depression, domestic difficulties, and inability to work, have a negative effect on life quality, irrespective of the pain itself.^1^,^9^

Unlike methods aimed at eliminating the cause of pain, or reducing the pain, rehabilitation programs mainly seek to improve patient function. When the objective is to improve function rather than to relieve pain, it is possible to evaluate rehabilitation outcomes more objectively.^10^–^12^

Primary or secondary ADL deficits are often seen in CLBPD. Primary ADL deficits are manifest when the functional deficit is more severe. They appear as difficulty or inability to care for the lower body, reducing the sitting time required for daily activities, and reducing the walking distance and the quality of walking, which may be slower, have reduced stride lengths, and involve reduced coordination between the thorax and the pelvis. Secondary ADL deficits may appear as reduction of the sitting time to enjoy a show or a movie, reduction of activities performed standing, difficulty in bending, and limitation of work capacity.

Most of the CLBPD rehabilitation programs that focus on functional improvement, often referred to as functional restoration programs, admitted outpatients with secondary ADL dysfunction. They used the percentage of patients returning to work and the change in sick leave days as outcome measures.^4^,^10^–^17^ Uncontrolled trials showed improved participation in work after rehabilitation programs, but controlled trials showed conflicting results.^12^,^13^ Several publications described rehabilitation of CLBPD with primary ADL deficit.^1^,^2^,^18^,^19^ Härkäpää and colleagues described an inpatient rehabilitation program, with better achievements than those of ambulatory rehabilitation. Few articles, however, describe effective rehabilitation of severe primary ADL deficit, and we did not find studies that compare rehabilitation effectiveness of patients with different grades of CLBPD severity, or studies showing whether rehabilitation can be effective even in patients with the most severe primary ADL deficit.^20^,^21^

To fill this gap, the present study retrospectively assessed the outcomes of an inpatient CLBPD rehabilitation program for patients with reduced performance on everyday life tasks (daily activities). It assessed the factors that affected program outcomes and compared them between levels of disability.

The objective of this study was to evaluate the extent of improvement in scores of performance of daily tasks in patients with primary ADL deficits during the CLBPD rehabilitation program. Secondary objectives were to evaluate whether or not severe disability affected this improvement and whether reduction of pain was reported during rehabilitation for the majority of patients.

## PATIENTS AND METHODS

### Patients

Data of 100 patients who were admitted consecutively to an inpatient rehabilitation program at Loewenstein Rehabilitation Medical Center were assembled for the purpose of the study. One of the patients was found to meet one of the exclusion criteria and was removed from the sample, leaving a sample of 99 patients. Inclusion criteria for the study, as well as for admission for the inpatient rehabilitation program, were a diagnosis of CLBPD, with deficit in primary ADL, despite standard care of a general practitioner and various specialists, including pain medication and individual physiotherapy, and an American Spinal Injury Association Impairment Scale (AIS) grade of D or E.^22^ Patients with an AIS grade of D were included only when their disability could not be explained by their mild spinal cord lesion (SCL) or the root lesion, and caregivers attributed it to chronic back pain. The types of functional deficits the patients had were inability to dress and wash their lower body, as well as limitations in sitting, standing, and walking. Exclusion criteria were spinal surgery within six months before admission to the rehabilitation program, and non-spinal medical conditions that disturb function (e.g. head injury).

### Procedure

All the patients included in the study underwent the same rehabilitation program, based on documented principles of CLBPD rehabilitation.^6^,^8^,^10^–^12^ The multidisciplinary team assessed and documented patients’ task performance at the start, during, and at the end of rehabilitation. The team, which included physiatrists, physiotherapists, occupational therapists, psychologists, and social workers, treated the various components of the CLBPD syndrome described in the introduction, in parallel.

Generally, patients were discharged from the inpatient rehabilitation program when the staff members’ qualitative assessment indicated no functional improvement for 2–3 weeks. Data for this study were collected by review of patients’ medical records for physical, social, and demographic variables, and for function and pain, that had been assessed on admission to the rehabilitation program and at discharge.

### The Rehabilitation Program

The rehabilitation program resembled inpatient CLBPD rehabilitation programs performed in few places in other countries,^20^ and it is likely unique in Israel. It included exposure to functional challenges of gradually increasing difficulty, such as bed mobility, transfers (from bed to chair, and to standing up) or dressing lower body with gradually decreasing assistance, sitting on an adapted chair for increasingly longer periods, or walking gradually increasing distances, with gradually decreasing support of aiding devices. It also included physical training for strengthening muscles and for improving endurance, ranges of motion, and fitness, as well as cognitive and behavioral interventions and counseling for domestic and social problems. In some cases, the program included ergonomic interventions. Treatment was delivered in individual and group sessions, at bedside, in the caregivers’ hospital offices, and at the physiotherapy and occupational therapy facilities of the hospital.

Alongside the efforts to alleviate pain and treat the various CLBPD components, caregivers received instruction on how to allay concerns and fears and express expectations for higher patient performance, irrespective of pain. Progress in functional missions was guided by a “contract,” with patients having to meet functional goals (e.g. tying shoelaces or walking a certain distance) on which they agreed with caregivers every few days, irrespective of pain. The rehabilitation hospital setting provided an environment that encouraged the improvement of function, in contrast to the natural environment, which may support dysfunction because of the fears and concerns of the patient and relatives.^19^

In addition, this setting allowed objective assessment of the patients’ physical ability while under 24-hour observation, including while in bed, in the bathroom, and in other circumstances not usually observed by caregivers in the ambulatory setting. This enabled continuous adaptation of the therapeutic program based on the patient’s observed abilities. It also allowed day-to-day follow-up of the patient’s response to analgesic medication and ongoing adaptation of medication type and dose for optimal pain control and maximum physical function during the rehabilitation program. This type of personalized care is not possible to administer in standard ambulatory outpatient rehabilitation.

### Outcome Measures

After data collection, a member of the research team translated the descriptions of ADL performance, which the medical treatment team had entered in the records of all the patients included in the study at the start and at the end of the rehabilitation program, into scores of the Spinal Pain Independence Measure (SPIM), for the quantitative assessment of function (higher scores represented better performance). The scale, which includes tasks in the areas (subscales) of mobility, activity in sitting and standing positions, and activity indoors (Table 1), was found to be reliable, valid, and responsive for CLBPD patients with deficits in primary ADL.^23^

**Table 1:** The Examined SPIM Subscales and Related Tasks.^23^

Subscale	Task*	Score†
Mobility		
	Mobility for short distances (indoors)	7

	Mobility for moderate distances (10–100 m)	7

	Mobility for long distances (more than 100 m)	7

	Stair management	5

Activity in sitting and standing positions		
	Activity in the sitting position	20

	Activity in the standing position	12

Indoor activity		
	Mobility in bed	6

	Transfers	6

	Washing lower body	6

	Dressing lower body	6

* Missing data: data for the tasks Maximum walking speed (maximum score=6) and Carrying loads (maximum score=12) were missing.

† The maximum score for the task.

We evaluated change in pain during rehabilitation using the patient records translated into the following scores, which research team members supported in a face validity process: worsening of pain, 0; no change, 1; minimal improvement, 2; slight improvement, 3; moderate improvement, 4; and major improvement, 5.

We also assessed the following demographic and clinical variables for their association with function or reported change in pain: age, gender, the presence of neurological deficit (AIS grade D), length of stay in rehabilitation (LOS), SPIM score at admission to the rehabilitation program, the change in SPIM scores between admission and discharge from rehabilitation (SPIM gain), opioid drug consumption and its daily dose (low dose, <20 mg oxycodone or 50 mg tramadol; moderate dose, 20–40 mg oxycodone or 50–150 mg tramadol; and high dose, >40 mg oxycodone or 150 mg tramadol), and the existence of an open compensation claim. The last-mentioned variable was examined because previous publications indicate that a compensation claim may affect functioning and the assessments of pain and disability.^24^,^25^

### Analysis

Data were expressed as mean and standard deviation (SD), or median, or as number and percentage. We used two-tailed *t*-tests to assess the differences in SPIM scores, reported changes in pain, and demographic or clinical factors between admission to the rehabilitation program and discharge, and between patient groups. We used a distribution-based method to assess the minimal important difference (MID) in SPIM scores (MID 0.5 SD).^26^ We used Spearman’s test to investigate the correlations between SPIM gain, admission SPIM score, and LOS, and between demographic and clinical factors and the perception of change in pain. We used analysis of variance (ANOVA), followed by Tukey’s test and a logistic regression to assess the influence of demographic and clinical factors on SPIM gain. To control for LOS, we included LOS in logistic regressions after square root transformation, as an independent variable. Data were analyzed using the Statistical Package for the Social Sciences (SPSS) version 27.0 for Windows (SPSS, Chicago IL, USA).

### Ethics

The study (CLBPD1L) was approved by the institutional review board (IRB) of Loewenstein Rehabilitation Medical Center (identifier 0005-09-LOE). As the study was retrospective and used anonymized data, getting an informed consent was waived. The investigators followed the ethical principles of the Declaration of Helsinki. The study conforms to STROBE cohort studies guidelines and reports the required information accordingly.

## RESULTS

### Patient Data

The mean age of the included 99 patients was 48.6 years (range 22–81; SD 13.8), 63 (63.6%) were men, and 75 (75.8%) were married. Twelve (12.1%) were employed before admission to the rehabilitation program, 76 (76.8%) were unemployed, and 11 (11.1%) were retired. The most commonly diagnosed spinal pathologies were spinal disc herniation, found in 58 patients (58.6%), spinal stenosis in 14 (14.1%), and spine injury in 31 (31.3%). Sixty-nine patients (69.6%) had AIS grade E, and 30 (30.3%) grade D. Background medical problems included metabolic diseases in 20 patients (20.2%), chronic lung disease in 19 (19.2%), hypertension in 19 (19.2%), and obesity in 18 (18.2%). Mean length of stay in rehabilitation (LOS) was 34.2 days (range 7–111; median 29; SD 20.4). The only opioid drugs that were documented in the records to have been used by participants were tramadol and oxycodone. The number of patients consuming these drugs increased from 21 (21.2%) at admission to 26 (26.2%) at discharge: 12 (12.1%) used a low dose, 8 (8.1%) a moderate dose, and 6 (6.1%) a high dose.

### Function

Data for the SPIM items “Maximum walking speed” (maximum score of 6) and “Carrying loads” (maximum score 12) were missing. Mean SPIM score was 48.7 as rehabilitation started, at admission to the rehabilitation program (range 0–82; SD 16.3), and increased to 57.8 at discharge, at the end of the program (range 22–82; SD 12.5, *P*<0.001; MID 8.1; effect size 0.6 SD). Seventy-one patients (71.7%) improved their daily task performance during rehabilitation: in 39 SPIM score gain was 9 points or more, and the highest SPIM score gain was 41 (Table 2).

**Table 2:** Functional Improvement During Rehabilitation.

SPIM Gain*	Number of Patients
<0 (reduced function)	1
0	27
1–10	38
11–20	20
21–41	13
>41	0

* SPIM gain = difference between discharge and admission SPIM scores.

**Figure 1:**
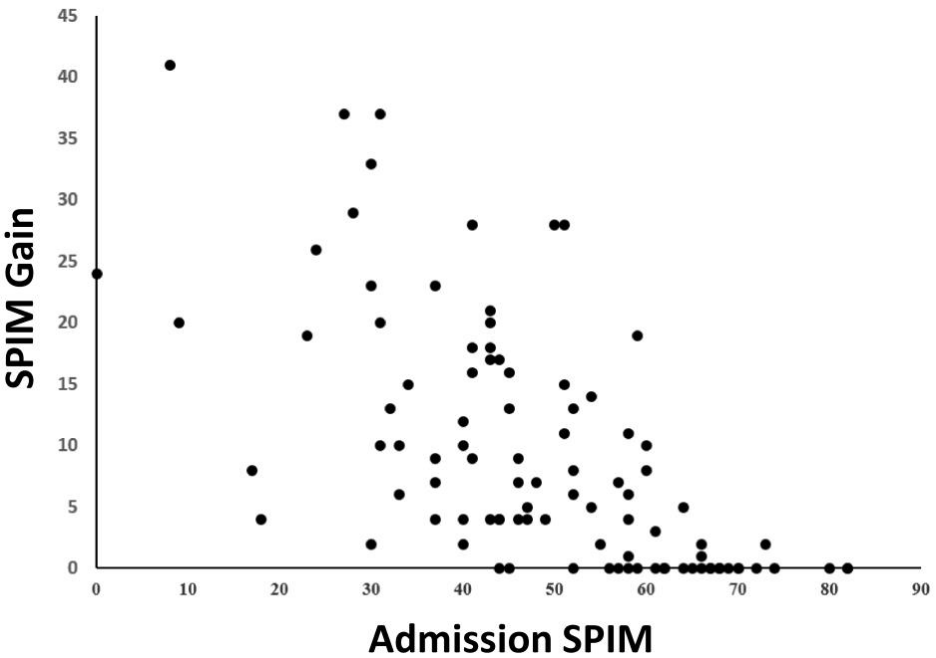
Severity of Disability and Rehabilitation Effect SPIM gain was higher in patients with lower admission SPIM score (*r*=−0.696, *P*<0.001).

The SPIM gain correlated negatively with SPIM score at admission to the rehabilitation program (*r*=−0.696, *P*<0.001; Figure 1). This correlation was independent of LOS: it persisted even after controlling for LOS (*P*<0.001), despite the fact that LOS was also negatively correlated with SPIM at admission (*r*=−0.620, *P*<0.001), and SPIM gain positively correlated with LOS (*r*=0.624, *P*<0.001; Table 3).

**Table 3:** Distribution of Patients and Functional Improvement According to the Length of Stay in Rehabilitation.

LOS, days	Number of Patients	SPIM Gain, Mean (SD)*
1–14	13	2.8 (7.9)
15–30	43	7.1 (8.9)
31–45	23	9.3 (8.8)
46–60	9	17.9 (9.5)
61+	11	16.4 (11.9)

* SPIM gain = difference between discharge and admission SPIM scores.

LOS, length of stay in rehabilitation; SD, standard deviation.

The negative correlation between SPIM gain and admission SPIM score had critical implications for admission SPIM scores of 50 or higher. Whereas SPIM gain was statistically significant (*P*<0.05) for patients with an admission SPIM score 50 or lower, it was non-significant for admission SPIM values above 50, and almost no functional improvement was achieved for patients with an admission score of 65 or higher (Figure 1).

There was no significant relationship between SPIM gain and age, gender, or presence of neurological deficit. The effect of opioid drug consumption on SPIM gain was found non-significant after controlling for LOS. The effect of an open compensation claim on SPIM gain after controlling for LOS was also non-significant.

### Reported Change in Pain

Patient records indicated that the reported pain decreased between admission to the rehabilitation program and discharge for 59 patients (59.6%). The decrease in reported pain was minimal for 24 patients (24.2%), slight for 13 (13.1%), moderate for 18 (18.2%), and major for 4 (4.0%). No pain relief was reported for 39 patients (39.4%), and worsening of pain for 1 (1.0%).

The reported pain reduction of patients with neurological deficit was smaller than that of patients without neurological deficit (*P*<0.05). A weak correlation was found between the number of patients using higher opioid dose at discharge and reported pain reduction (*r*=0.211, *P*<0.05). The LOS of patients who were consuming opioids (mean LOS 51.9 days) was significantly higher than that of those who were not (mean LOS 33.0 days, *P*<0.001). There was no significant difference, however, in age and in the change in pain between patients who did and who did not consume opioids.

Age, gender, LOS, SPIM score at admission, SPIM gain, and an open compensation claim were not significantly associated with the reported change in pain.

## DISCUSSION

Evaluating the outcomes according to the primary and secondary objective of the study, we found that daily performance scores increased during the rehabilitation program, that the increase was more prominent in patients with the most severe disability, and that most patients reported a decrease in pain.

Mean SPIM score, which reflects the performance of primary ADL tasks relevant for CLBPD, increased significantly between admission to the program and discharge. The increase in SPIM score was evident in 72% of participants (Table 2). In 39% of the participants SPIM score increase was 9 points or more, which suggests a meaningful improvement in these participants’ functionality and quality of life. This demonstrated the effectiveness of the rehabilitation program in reducing CLBPD-related primary ADL deficits.

The functional change during rehabilitation was negatively associated with the severity of disability, irrespective of program duration (LOS), and the other factors examined in the study did not affect it. The improvement in SPIM scores was significant in patients with an admission SPIM score of 50 or lower, but not in those with a score of 51 or higher (Figure 1). These findings suggest that the effects of the inpatient rehabilitation program manifest primarily in patients with the most severe CLBPD.

Most of the patients required 2–6 weeks to reach a functional plateau, but some gradually improved over a longer period, and others showed a satisfactory performance within a shorter period (Table 3).

Functional improvement was consistent with that in some rehabilitation programs, which also showed effectiveness in improving daily functioning, or other functional aspects, although other programs showed either no improvement or poor results.^2^,^11^–^13^,^15^–^19^,^21^,^27^ Unlike most other programs, however, the rehabilitation program presented here focused on primary ADL. This may explain the different effects of age and gender compared to previous reports, which focused on vocational outcomes and pain.^15^ Other factors studied previously, which affect vocational outcomes mainly, were not addressed in the present study because they appear to be less relevant for the purposes of the present program.^12^

The study also showed pain reduction in 60% of participants. Although pain reduction was minor-to-moderate for the majority of patients, it can be considered satisfactory for the patients included in this study, who had prolonged severe LBP and were resistant to many previous treatments. Moderate evidence of improvement in pain was also found in other multidisciplinary rehabilitation programs, although some rehabilitation programs did not result in improvement.^12^–^14^,^16^,^27^

The lack of correlation between pain reduction and function at admission to the rehabilitation program, and between pain reduction and functional gain suggests that pain alone does not explain the disability in CLBPD patients, and supports the importance of functional restoration in CLBPD. This lack of correlation is consistent with findings in previous studies of functional outcomes at work or in daily activities.^12^,^15^

The correlation between pain reduction and opioid use is not surprising, and the increase in opioid consumption during rehabilitation reflects efforts to achieve pain relief. The weakness of this correlation, however, as well as the moderate dose required compared with other CLBPD rehabilitation programs, and the similarity in age and reported pain reduction between those who did and did not consume opioid drugs, further support the notion that the contribution of pain alone to CLBPD is minor.^28^–^31^

Lack of correlation between functional improvement and neurological deficit supports the attribution of the disability to chronic back pain and not to SCL in patients who were included with an AIS grade of D.

The failure of this study to show an effect of an open compensation claim on outcomes is important because open compensation claims may lead to postponing the rehabilitation of patients with CLBPD until the claim is settled, which may reduce patients' functional achievements.^15^,^24^,^25^ This can happen because a compensation claim is frequently regarded as an incentive for primary gain, which may bias assessments of pain and disability, and reduce the patient’s motivation to improve functioning. Physicians and administrators who believe that reported pain or disability is exaggerated by a primary gain may be reluctant, therefore, to decide on admissions or referrals to rehabilitation before the claim is settled. Our findings, which imply that the existence of an open compensation claim did not affect pain and disability assessments and did not impede functional improvement during rehabilitation, as well as findings of a previous study, dispute postponing rehabilitation until the claim is settled.^25^

### Study Limitations

Study limitations include the retrospective use of recorded data and the lack of long-term follow-up. They also include the missing data for the SPIM items “Maximum walking speed” and “Carrying loads,” which reduced the total SPIM score by up to 18 points and may have affected SPIM gain. The scoring of these items may not have affected the main findings of the study but may have reduced the difference in SPIM gain between patients with more severe and lighter disability because fast walking and carrying loads are probably more demanding than other SPIM tasks. Another limitation is that the SPIM scores were calculated by the research team and not the medical treatment team, which may have caused inaccuracy in the assessment of SPIM. This could bias the values of SPIM gain presented in the study because the timing of discharge, which may affect SPIM gain, was not based on the SPIM scores. This potential inaccuracy, however, had an equal chance of increasing or decreasing SPIM score. Therefore, the mean differences between the assessments of the clinical and research teams in the studied groups were likely to be very small. These differences were also likely to appear at both the start and the end of rehabilitation. The effect of this limitation on SPIM gain, therefore, was probably negligible.

## CONCLUSION

The inpatient multidisciplinary chronic LBP rehabilitation program was found to be effective in restoring daily task performance and provided limited pain relief. This study, which examined for the first time rehabilitation outcomes in patients with the most severe CLBPD, showed that rehabilitation was most effective for patients with the most severe disability. The findings support the precedence of function over pain in CLBPD, can be generalized to other severe CLBPD patients, and do not support postponing admission to a rehabilitation program while a compensation claim is open. We suggest that additional rehabilitation departments consider inpatient rehabilitation programs to achieve optimal improvement in patients with severe CLBPD.

## Abbreviations

ADL: activities of daily living
AIS: American Spinal Injury Association Impairment Scale
ANOVA: analysis of variance
CLBPD: chronic low back pain disability
LBP: low back pain
LOS: length of stay in rehabilitation
MID: minimal important difference
SCL: spinal cord lesion
SD: standard deviation
SPIM: Spinal Pain Independence Measure.
